# Social conformity is a heuristic when individual risky decision-making is disrupted

**DOI:** 10.1371/journal.pcbi.1012602

**Published:** 2024-12-02

**Authors:** Mark A. Orloff, Dongil Chung, Xiaosi Gu, Xingchao Wang, Zhixian Gao, Guiding Song, Chandana Tatineni, Shuai Xu, Brooks Casas, Pearl H. Chiu

**Affiliations:** 1 Fralin Biomedical Research Institute at VTC; Virginia Tech; Roanoke, Virginia, United States of America; 2 Graduate Program in Translational Biology, Medicine, and Health; Virginia Tech; Roanoke, Virginia, United States of America; 3 Center for Mind and Brain; University of California Davis; Davis, California, United States of America; 4 Department of Biomedical Engineering; UNIST; Ulsan; South Korea; 5 Department of Psychiatry, Department of Neuroscience; Icahn School of Medicine at Mount Sinai; New York, New York, United States of America; 6 Department of Neurosurgery; Beijing Tiantan Hospital affiliated to Capital Medical University; Beijing; China; 7 Methodist Family Medicine Residency in Dallas; Dallas, Texas, United States of America; 8 Department of Psychology; Virginia Tech; Blacksburg, Virginia, United States of America; Ecole Normale Superieure, FRANCE

## Abstract

When making risky choices in social contexts, humans typically combine social information with individual preferences about the options at stake. It remains unknown how such decisions are made when these preferences are inaccessible or disrupted, as might be the case for individuals confronting novel options or experiencing cognitive impairment. Thus, we examined participants with lesions in insular or dorsal anterior cingulate cortex, key regions implicated in risky decision-making, as they played a gambling task where choices were made both alone and after observing others’ choices. Participants in both lesion groups showed disrupted use of standard utility-based computations about risky options. For socially situated decisions, these participants showed increased conformity with the choices of others, independent from social utility-based computations. These findings suggest that in social contexts, following others’ choices may be a heuristic for decision-making when utility-based risk processing is disrupted.

## Author summary

When humans make decisions in social situations, we typically weigh both our individual preferences and information about what others choose. There are also common circumstances where individual preferences may be unavailable (e.g., when encountering new options or for individuals with certain neuropsychiatric conditions). To understand how choices are made in a social context when individual preferences are unavailable, we evaluated individuals with lesions in brain regions that encode risk preferences while these individuals made risky choices both independently and after viewing others’ choices. We find that when individual risk preferences are not computed, participants use a cognitive shortcut of making the same choices that others make. This shortcut may explain the heightened influence of others on the choices of some vulnerable individuals.

## Introduction

In social contexts, decision-makers sometimes follow a crowd despite their own preferences toward a different course of action [1,2]. This phenomenon has been documented in various forms in humans [3] and in nonhuman primates [4]. Recent studies indicate that when making socially situated choices, humans combine individual preferences for the options at stake with individual valuation of social information; contributions from each of these determine when choices made in a social context diverge (or not) from that which one’s preferences outside a social context would predict [5–11]. These previous studies have largely focused on understanding how social information contributes to choices with respect to individuals’ preferences about risk.

Here, we investigated how risky decisions in social contexts are made when utility-based risk preferences are disrupted or inaccessible, as might be the case for individuals with certain psychopathologies or who are confronting novel options (e.g., Gershman & Niv [12]). Specifically, previous literature on decision-making in humans and non-human primates indicates that when individuals have a limited amount of private information, they tend to use various cognitive shortcuts or heuristics (e.g., mimicking other agents) to simplify decision processes [13–16]. In the current study, we use a risky decision-making task and examine whether such heuristics facilitate socially situated decision-making about risk when utility processing is disrupted.

Previous work has shown that individual preferences and information from others are combined during risky decision-making under social influence [7,8,11,17]. Neurally, the insular and dorsal anterior cingulate cortices (dACC) have been shown to encode an interaction between one’s own risk preference and information from others. As such, these regions show greater hemodynamic responses when an individual’s preference differs from the observed choices of others [8]. The insula and dACC have also been consistently implicated in decision-making about risky options in non-social contexts, via lesion (insula only), functional neuroimaging (insula and dACC), and electrophysiology (insula) studies [18–23]. Thus, in situations where the structural or functional integrity of these regions is disrupted (e.g., in some neurological or psychiatric disorders) or where insufficient information or differing abilities impose constraints, individual computations and decisions about risky options may be disrupted, leaving open the question of how socially informed decision-making occurs in those cases. To examine this question, we administered a gambling task in both social and non-social contexts to individuals with focal insula or dACC lesions and tested models that measured the degree to which utility-based or heuristic-based processes were implemented during decision-making.

**Fig 1:**
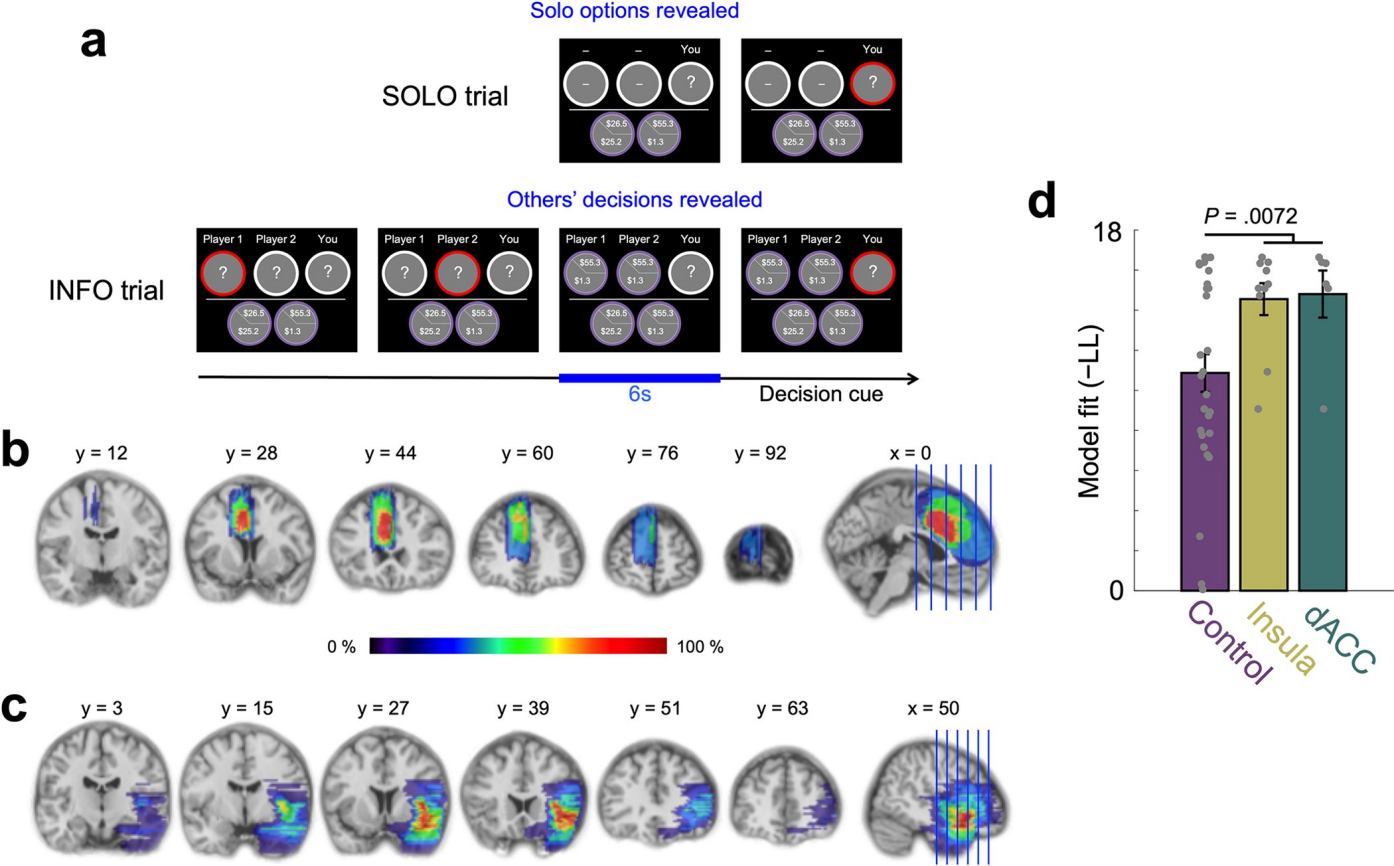
Experimental paradigm and lesion reconstruction. **(a)** Participants made a series of choices between one ‘safer’ gamble and one ‘riskier’ gamble. On each trial, participants viewed a new pair of gambles. On some trials, participants made choices alone (Solo trials). On other trials, they were asked to make choices after observing two other players’ choices (Info trials). The Info trials had three different types based on the composition of the two other players’ choices: ‘safe, safe,’ ‘risky, risky,’ and ‘mix.’ Four trial types (Solo, Info: ‘safe’, Info: ‘risky’, and Info: ‘mix’) were intermixed. Reconstructions of **(b)** dACC (N = 6) and **(c)** insula (N = 10) lesions are shown. The color bar represents the degree of lesion overlap among patients. To show the lesion overlap across participants, all lesions are shown overlaid on one hemisphere. **(d)** Power utility model fit for Solo trials as measured by negative log likelihood (−LL; lower values indicate a better fit) shows that individuals with insula or dACC lesions had significantly worse model fit than non-lesion control participants (NCs; *P* = 0.0072; NC vs insula: *P* = 0.032; NC vs dACC: *P* = 0.073), suggesting disrupted utility-based risky decision-making in participants with insula or dACC lesions; see also S1 Fig for model-agnostic data consistent with disrupted sensitivity to risk in lesion participants).

Individuals with focal insula (N = 10) or dACC lesions (N = 6) and non-lesioned control participants (NCs; N = 28) made a series of choices between pairs of gambles alone (‘Solo’ trials) and after observing two others’ choices (‘Info’ trials; Fig 1A–1C; task developed in our previous work [7,8]). Trials comprised four distinct types: Solo, where participants did not see others’ choices; Info: ‘safe’, in which both of the two social others’ choices that the participant observed were the safer (i.e., lower payoff variance) gamble; Info: ‘risky’, in which the two social others’ choices were the riskier (i.e., higher payoff variance) gamble; and Info: ‘mix’, where one social other’s choice was the safer gamble, and one other’s choice was the riskier gamble. Info and Solo trials were intermixed throughout the task.

## Results

### Disrupted utility-based decision-making about risky options

To first evaluate non-social decision-making under risk in our participants, we examined how well a standard utility model (i.e., power utility function [24]) and softmax choice rule [25]; see Materials and Methods for model specifications) explained behavioral choices in Solo trials. Per economic utility theory [24], individuals’ preferences about risk can be captured by the concavity (ρ) of power utility functions (U(*x*) = *x*^ρ^) that reflect individuals’ utility computations about risky options. We thus used a goodness-of-fit metric of the power utility model for explaining individual participants’ decisions about risky options as an indication of the extent to which utility-based decision-making was used. Specifically, we used the median negative log likelihood as it reflects the fit at the median parameter estimates which we use throughout the paper (see **Methods: Behavioral model specifications and model comparison**). As expected, participants with insula or dACC lesions showed worse model fit compared with NCs [i.e., comparisons of negative log likelihood (−LL) in Solo trials, *P* = 0.0072; see also [18–21] reporting insula or dACC involvement in utility-based decision-making under risk. This remained true after controlling for individuals’ risk preference (*P* = 0.0019). Post-hoc analyses by lesion area indicated that compared with NCs, participants with insula lesions showed worse model fit while participants with dACC lesions trended toward worse model fit (*P =* 0.032 and *P* = 0.073, respectively; Fig 1d; see Materials and Methods for full details and S1 Fig for model-agnostic choice behaviors). Participants with insula lesions and participants with dACC lesions did not differ in model fit (*P* = 0.87, Bayes factor (BF_null_) = 2.27).

### Socially situated decision-making about risk

**Fig 2:**
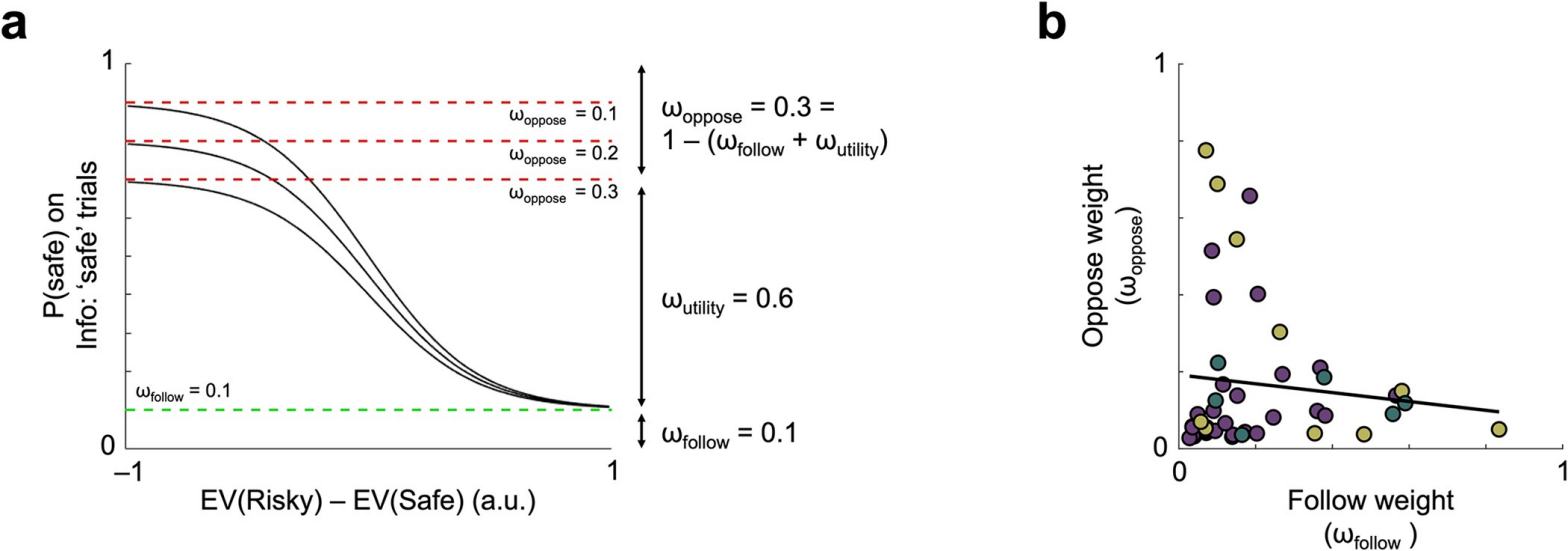
Hybrid social decision-making model visualization and explanation. The hybrid model uses two weights (ω_follow_ and ω_oppose_) to represent utility–independent contributions of social information to participant decisions, indexing the degree to which participants follow or oppose the choices of social others, independent from utility-based risk processing. **(a)** Effectively, the ‘follow’ and ‘oppose’ weights act to constrain the probability of choosing a given option. On Info: ‘safe’ trials, the maximum probability of choosing the safe option (regardless of any gamble information) is equivalent to 1 –ω_oppose_ (shown as the dotted red lines for different ω_oppose_ values) while the minimum probability of choosing the safe option (regardless of any gamble information) is equivalent to ω_follow_ (shown as the dotted green line). Likewise, on Info: ‘risky’ trials (not depicted), the maximum probability of choosing the safe option is 1 –ω_follow_, and the minimum probability of choosing the safe option is ω_oppose_. The black lines represent the other-conferred utility process, setting the probability of choosing the safe option as a function of gamble information transformed by one’s risk preference (according to a power utility function), sensitivity to utility (softmax choice rule), and subjective value conferred by others choosing a particular option (other-conferred utility; OCU). **(b)** In constructing this model, we remained agnostic as to whether oppose and follow represent dissociable processes. Nonetheless, after model estimation, we further tested if there was a relationship between these oppose and follow processes. We find no significant correlation between ω_follow_ and ω_oppose_ when measured across all participants (Pearson’s r = –0.11, *P* = 0.47, robust correlation, BF_null_ = 6.52), suggesting dissociable processes for individuals’ decisions to follow or oppose social others’ choices. Each point represents an individual participant, and lines are the regressions between the indicated parameters.

Using these participants with insula or dACC lesions as exemplars, we next sought to examine whether heuristics may facilitate socially situated decision-making when utility-based risk processing is disrupted. Previously, we developed an ‘other-conferred utility (OCU)’ model and showed that the impact of social information on decisions about risky options is captured by increased utility to the option chosen by others and thus, increased likelihood of choosing this option [8]; we refer to this type of social information use as ‘OCU-based’ or ‘utility-based.’ This model, along with its modified version, has been validated in independent simulations showing both model and parameter recovery [7,26]. Here, to accommodate the possibility of incorporating social information into decisions independent of utility computations (i.e., ‘heuristic-based’ or ‘OCU-free’ use of social information), we extend our previous OCU model [8] and introduce weight parameters that allow a mixture between OCU-based and OCU-free contributions of social information to participants’ decisions (‘hybrid model’ hereafter). The OCU-free parameters allow the initial testing of zeroth order social heuristics that have been previously shown to affect decision-making [13]; in the present work, these choices are either in alignment with or in opposition to the choices made by others [8,9]. We subsequently call these choices that ‘follow’ or ‘oppose,’ respectively, those of social others. In this mixture model, weight parameters ω_utility_, ω_follow_, and ω_oppose_ capture the degree to which social information is incorporated into decision-making, dependent upon (ω_utility_) or independent from (ω_follow_ and ω_oppose_) social utility-based processes (Figs 2, S2, S3, and S4 for further model description and validation information). Note that if an individual uses social information in a way that fully relies on other-conferred utility (i.e., ω_utility_ = 1), the mixture model prediction is equivalent to that of the original OCU model [8]; if an individual uses social information in a manner that is fully free from other-conferred utility (OCU-free; i.e., ω_follow_ + ω_oppose_ = 1), their choices would be consistent with the simple heuristics of fully following or opposing those of social others, regardless of the gamble options. See S5 Fig for model comparison of the hybrid model against an only OCU-free heuristic model, an only OCU-based model, and a Solo risk preference model, showing best fit of the hybrid model for explaining participants’ choices.

**Fig 3:**
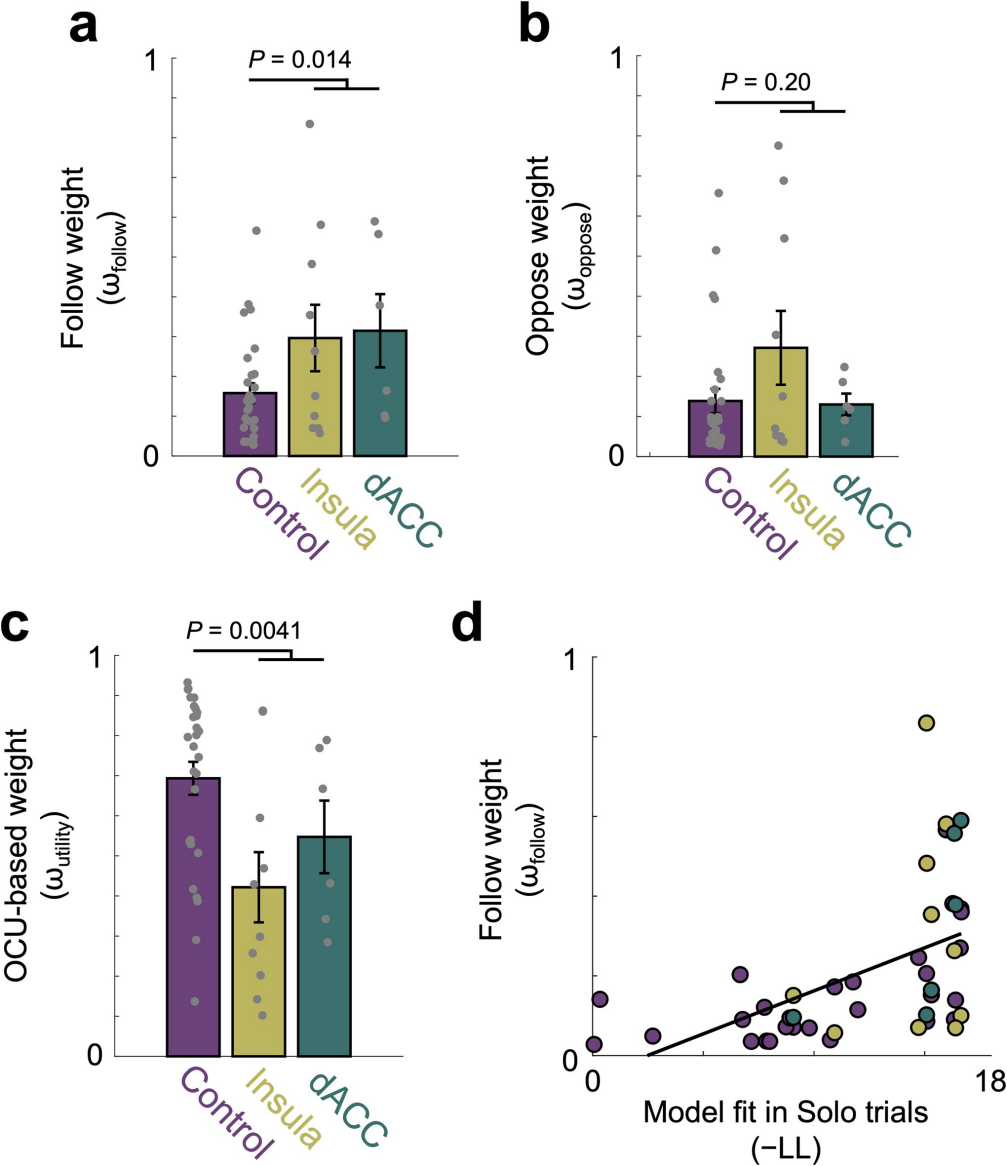
Model-derived results show increased social following in individuals with disrupted utility-based risky decision-making. **(a)** Parameter estimates across all trials showed that individuals with insula or dACC lesions had significantly larger ω_follow_ estimates than non-lesioned control participants (NC; NC vs insula: *P* = 0.036; NC vs dACC: *P* = 0.025; dACC vs insula: *P* = 0.89, BF_null_ = 2.28), indicating that the lesion participants were more likely to conform with others’ choices during decision-making in a social context. **(b)** The insula and dACC lesion groups’ ω_oppose_ estimates were comparable to NC estimates. **(c)** NC participants had larger ω_utility_ estimates than individuals with lesions (*P* = 0.0041). Post-hoc analyses revealed that the difference was specific to individuals with insula lesions (NC vs insula: *P* = 0.0043; NC vs dACC: *P* = 0.14, BF_null_ = 1.17; dACC vs insula: *P* = 0.36, BF_null_ = 1.71). **(d)** We tested a link between decreased utility-based risk processing (measured by negative log likelihood in Solo trials; −LL) and increased ω_follow_. Across all groups, individuals with worse utility-model fit (larger −LL) in Solo trials showed greater ω_follow_ (Pearson’s r = 0.52, *P* = 2.7e−04, robust correlation).

Compared with non-lesion controls, participants with insula or dACC lesions as a combined group showed significantly larger following weights (ω_follow_; Fig 3A; *P* = 0.014). This group difference result remained consistent after controlling for individuals’ risk preference (*P* = 0.0074). Post-hoc analyses by lesion area indicated that relative to the non-lesion controls, participants with insula lesions (*P* = 0.036) and with dACC lesions (*P* = 0.025) also both showed significantly larger ω_follow_. These results indicate that individuals with focal lesions in insula or dACC implement a ‘follow others’ choices’ heuristic during decision-making in a social context, which behaviorally manifests as increased conformity with the choices of others.

Model-agnostic measures of participants’ tendency to follow the choices of others were correlated with the model-derived ω_follow_ (S2C and S2D Fig; for complete mixture model details, see S2A Fig). Tendencies for choices to oppose those of social others (captured in the ω_oppose_ parameter) did not differ between participants with insula or dACC lesions and controls (Fig 3B, *P* = 0.20, BF_null_ = 1.64). A post-hoc analysis additionally shows comparable ω_oppose_ parameters between insula lesion and dACC lesion groups (*P* = 0.28, BF_null_ = 1.48). As we hypothesized, participants with insula or dACC lesions, relative to non-lesion control participants, had diminished ω_utility_ (Fig 3C; *P* = 0.0041), indicating less use of utility-based risk processing. This remained the case after controlling for individuals’ risk preference (*P* = 0.0018). Post-hoc analyses by lesion area indicated that participants with insula lesions had diminished ω_utility_ (*P* = 0.0043); participants with dACC lesions did not differ from control participants or participants with insula lesions (dACC vs NCs: *P* = 0.14, BF_null_ = 1.17; dACC vs insula: *P* = 0.36, BF_null_ = 1.71). See S3 Fig for individual ω estimates and further examination of model weights.

If individuals with impairments in utility-based risky decision-making indeed rely on conformity to a greater extent, we may expect a parametric relationship between degree of impairment and use of the ‘follow others’ choices’ heuristic. In this case, across both lesion and control groups, individuals who are most disrupted in utility-based risky decision-making would be expected to show greater use of this heuristic when making decisions under risk in a social context. To evaluate this possibility, we used model fit (negative log likelihood; −LL) calculated for choices in Solo trials (data presented in Fig 1D) as a measure of disruption in utility-based risky decision-making and examined the association of model fit with individuals’ model-derived following weights (ω_follow_). Across all three groups, individuals’ tendencies to follow the choices of social others (ω_follow_) were significantly correlated with greater −LL in Solo trials (greater −LL indicating more disrupted utility-based risk processing; Pearson’s r = 0.52, *P* = 2.7e−04, robust correlation; Fig 3D). This relationship was consistent after controlling for individuals’ risk preference (Pearson’s r = 0.52, *P* = 3.0e−04, robust correlation). That is, individuals whose utility-based risky decision-making was most disrupted conformed the most with the choices of others. This relationship was replicated in an independent sample of control participants (S5B Fig), providing convergent evidence supporting the notion that there is a trade-off between utility-based processes and cognitively less demanding heuristics that facilitates decisions about risky options in a social context.

## Discussion

Humans use social information in different ways to guide decision-making [27,28]. The present results provide a model-based explanation for how in social situations, individuals with disrupted utility-based risk processing may make choices about risky options. Using individuals with insula or dACC lesions as exemplars, we show that these individuals exhibit disrupted utility-based risk processing and use social information independent from utility computation to guide decisions about risky options. These findings suggest social conformity can serve as a heuristic alternative to using social information in a utility-based way during socially situated decision-making when individuals’ risk processing is impaired.

In previous studies using the same task, we have shown that when making decisions under risk, individuals combine social information with personal preferences and do so by adding other-conferred utility to the option that others choose [7,8]. Here, we identify an alternative heuristic path of using the same type of social information when personal preferences are not computed. This discovery was made possible by examining participants with lesions in insula and dACC (regions that have been consistently implicated in functional neuroimaging studies of risk processing) as exemplars of individuals with disrupted risk processing [18,19,29,30]. Our post-hoc analyses examined whether insula and dACC participants differed with regard to disruptions in risk processing; we found no evidence of group differences between insula and dACC participants in power-utility model fit to their choices on Solo trials. While to our knowledge, this work is among the first to show evidence of disrupted risk processing in participants with dACC lesions, it is perhaps unsurprising, given that dACC and insula frequently co-activate in functional neuroimaging studies of risk-related decision-making [18,19,29,30]. We also considered our work in the context of prior work implicating dMFC (often extending into dACC) in social processes [7,26,31–33]; while we did not observe social-specific differences in our dACC vs insula participants, this is an area of relevant future study.

We emphasize that the ‘follow others’ choices’ heuristic identified in the current study is consistent with a ‘copy-when-uncertain’ strategy previously demonstrated in perceptual decision-making, such that individuals are more likely to rely on the behaviors of others when they are less confident about their own decisions [34–36]. The present study adds to this literature by demonstrating that a copy-when-uncertain strategy extends to socially situated decision-making about risky options. Of note, although a ‘follow others’ choices’ heuristic seems qualitatively different from utility-based use of social information, one framework that captures both paths is a Bayesian-like update framework, in which individuals use social information to update their likelihood of choosing the safe gamble [37–40]. That is, the utility difference between gamble options may serve as a Bayesian prior, updated by novel information contained in social others’ choices. The extent to which an individual combines their priors with the novel social information depends on the *relative* uncertainty between the gamble utility difference and the social information where the information with lower uncertainty contributes more to the final decision (i.e., the posterior). In this framework, individuals who have disrupted utility-based risky decision-making (e.g., the lesion group herein) would be expected to have a relatively flat prior for the utility difference of gamble options. Starting with this flat prior, a Bayesian process would indicate that any subsequent choices made under social influence would be primarily driven by the social information because the social information carries more certainty than does the individuals’ computed utility difference between the choices at hand. By comparison, individuals with intact utility-based risk processing (e.g., NCs) would have relatively sharper priors for utility differences between gambles and the social information would subsequently carry less impact for participants’ decisions.

Broadly, these results have important implications for circumstances associated with suboptimal risky decision-making, such as during neurodevelopment in adolescents [41,42] or in individuals with neurological and psychiatric disorders characterized by altered decision-making [43–46]. This disrupted utility-based risk processing may in-turn play a role in the particularly prominent influence of social others in adolescent decision-making [47,48] and also in the onset and maintenance of substance misuse and related psychopathology [49,50]. In these cases, disruption to utility-based risk processing may lead to heightened influence of other people in socially situated decision-making. The presence of heuristic and utility-based processes for incorporating information from social others into risky decision-making thus suggests distinct avenues for addressing maladaptive decision-making (e.g., enhancing preferences vs changing social group composition).

The limitations of the present study suggest some avenues for future investigations. First, although the information being shown to participants in this study is social information (i.e., choices of others), it is possible that influences on participant choices may be due to a more general visual or information effect, whereby merely seeing other information (regardless of its source) may influence behavioral choices. While we previously demonstrated in non-lesioned adults using the same task that effects on participants’ behavior was indeed ‘social’ (i.e., no influence on participants choices were observed in a non-social visually controlled experiment; Supplemental Fig 8 from [8]), this may not extend to individuals with insula and/or dACC lesions. Further work is necessary to test whether the ‘follow others’ choices’ effect when risk processing is disrupted is specific to social information or more general to any type of information. Second, we combined participants with dACC or insula lesions into a single ‘lesion group’ because of the common role of these regions in risk processing [18–23] and to maximize power for our primary intention of examining socially situated decision-making under risk. Still, it is worth noting the unique roles of these regions in other cognitive processes. For example, dACC is strongly implicated in social cognition [28] and social learning [51], while insula is more strongly implicated in interoception [52]. Future studies with larger samples ought to examine specificity of foci in these regions against more nuanced decision-making models and distinct social cognitive processes. We might expect relatively stronger impairment in social information processing in individuals with dACC lesions (and potentially stronger use of heuristics), whereas we might see stronger effects of risk processing (where interoception is thought to be a key component [53]) or dissociation of different risk models (e.g., [54]) in those with insula lesions. Third, we note that the perceptual ‘copy when uncertain’ strategy has exceptions (e.g., [55]) and similarly, the use of a ‘follow others’ choices’ heuristic may not be universally implemented in individuals for whom utility-based risk preferences may be disrupted, inaccessible, or otherwise not present (e.g., during substance misuse, individuals with certain psychopathologies, adolescents confronting novel options). We look forward to future studies examining these possibilities. Lastly, we point out that the model comparison analyses indicate that the Hybrid model was not well dissociable from the OCU-based, OCU-free, and Solo RP models, perhaps due in part to the nested nature of these models or the presence of multiple overlapping processes during socially-situated decision making about risky options. While our primary findings are based on robust parameter estimates of heuristic processes within the Hybrid model, the model comparison analyses indicate that the Hybrid model alone is unlikely to capture the range of social influences on risky decision-making; future studies are necessary to implement study designs that allow these processes to be detangled (see S4 Fig for further discussion).

Social contexts affect the decisions we make [56] and information received from others governs if and how one’s decisions are influenced by social others [57, 8]. Administering a risky decision-making task to participants with insula or dACC lesions revealed contributions of social information to decision-making that are independent from those relying on individuals’ utility-based computations about risk. Our model-based analytic approach and lesion data point to social conformity as a heuristic alternative in the absence of deliberative utility-based computation, during socially-situated decision making about risky options.

## Materials and methods

### Ethics statement

All participants provided written informed consent and were paid for their participation. The study was approved by the Institutional Review Boards of Virginia Tech and Tiantan Hospital of Capital University of Medical Sciences, Beijing, China.

## Experimental model and subject details

### Participants

Forty-six neurologically intact non-lesioned control (NC) subjects (male/female = 27/19, age = 44.28±7.63), 11 with focal insula lesions (male/female = 5/6, age = 44.00±11.85), and 13 with focal dorsal anterior cingulate cortex (dACC) lesions (male/female = 8/5, age = 38.38±11.43) participated in the current study. All participants reported no previous or current psychiatric conditions; all controls reported no previous or current neurological conditions. All participants with lesions were recruited from the Patient’s Registry of Beijing Tiantan Hospital, Beijing, China. NCs were recruited in local Beijing communities and were matched with patients for age, sex, and education.

Participants who showed choices that met a priori–defined behavioral exclusion criteria were excluded from all analyses. Specifically, 11 NCs, one participant with an insula lesion, and three participants with dACC lesions who chose the option with greater high payoff value less frequently as the probability of winning increased (comparing average gamble choices between winning probabilities of 40% to 90%) were excluded. Seven NCs and two participants with dACC lesions who always chose the safer or riskier gamble on all 24 Solo trials were additionally excluded. These exclusionary criteria are necessary to ensure that there are enough trials for accurate parameter estimation and that the assumptions underlying model estimation are met. We note that we excluded these participants from estimation because for participants who choose all risky or all safe, bi-directional social influence is not possible. That is, participants who always chose the risky option cannot be influenced to make riskier choices; similarly, participants who always chose the safe option cannot be influenced to make safer decisions. Finally, two additional participants from the dACC lesion group whose lesions were not localized within the dACC region were excluded from all analyses. Therefore, the analyzed sample included 28 NCs (male/female = 17/11, age = 42.82 ± 8.09), 10 participants with insula lesions (male/female = 4/6, age = 43.10 ± 12.09), and 6 participants with dACC lesions (male/female = 4/2, age = 34.33 ± 6.74; see S7 Fig for evaluation and confirmation of our main results across different exclusion criteria and further discussion).

Groups were well matched in sex (*P* = 0.59; NC vs insula: *P* = 0.28; NC vs dACC: *P* = 0.80; dACC vs insula: *P* = 0.34), lesion laterality (*P* = 0.54), education (*P* = 0.18; NC vs insula: *P* = 0.18; NC vs dACC: *P* = 0.34; dACC vs insula: *P* = 0.97), days since surgery (*P* = 0.37), lesion size (*P* = 0.92), and handedness (*P* = 0.74; NC vs insula: *P =* 0.44; NC vs dACC: *P* = 0.14; dACC vs insula: *P* = 0.12). Age was comparable between lesion and control groups (*P* = 0.31), between NCs and participants with insula lesions (*P* = 0.93) and between the two lesion groups (*P* = 0.13), whereas those with dACC lesions were significantly younger than NCs (*P* = 0.024). The lesion group had significantly lower Mini-Mental State Examination (MMSE [58]) scores than NCs (*P* = 0.0005; NC vs insula: *P* = 0.0013; NC vs dACC: *P* = 0.0091). However, the two lesion groups had comparable MMSE scores (*P* = 0.97). Because MMSE and age were different between at least one lesion group and NCs, we examined whether disrupted utility-based risky decision-making (Fig 1D) still differed between the lesion group and NCs after accounting for variation due to these demographics and whether the correlation between model fit and social heuristic remained significant (Fig 3D). Specifically, we first regressed out age or MMSE in separate multiple linear regression models and then performed either a group comparison or robust correlation test on those residuals to confirm lesion group or omega follow, respectively remained a significant predictor of disruption in utility-based risky decision-making. Indeed the lesion group was a significant predictor of disruption in utility-based risky decision-making after regressing out either age (*P* = 0.0072) or MMSE (*P* = 0.0094) and omega follow was significantly correlated with disruption in utility-based risky decision-making after regressing out either age (r = 0.46, P = 0.0016) or MMSE (r = 0.43, P = 0.0038) (Table 1).

**Table 1:** Participant characteristics.

	Non-lesion control (N = 28)	Insula lesion (N = 10)	dACC lesion (N = 6)
Male/Female	17/11	4/6	4/2
Age (years)^a^	42.82 ± 8.09	43.10 ± 12.09	34.33 ± 6.74
Handedness (R/L)	(27/1)	(10/0)	(5/1)
Education (years)	12.43 ± 1.95	11.4 ± 2.37	11.33 ± 4.63
MMSE	29.57 ± 0.79	28.30 ± 1.42	28.33 ± 1.51
Days since surgery	—	555.90 ± 560.31	815.33 ± 520.95
Laterality (R/L)	—	5/5	4/2
Lesion size (ml)	—	44.93 ± 34.12	46.89 ± 41.73

L, left; R, right; MMSE, Mini-Mental State Examination [58]; ^a^The age at the testing date; Regression analysis confirmed that our main findings were not affected by group demographic differences (see Materials and Methods for statistical comparison details). Data are represented as mean ± SD.

## Method details

### Lesion reconstruction

Lesion reconstruction was performed by a research assistant who was blind to the study design and behavioral results (C.T.) and was confirmed by a senior researcher (X.G.). In brief, lesions evident on T1- and T2- weighted MRI scans were identified and transcribed onto corresponding sections of a template to create a volume of interest (.voi) file. The template was derived from an MRI volume of a normal control (ch2.nii) created by Christopher Rorden (University of South Carolina, Columbia, SC) and provided for use with MRIcron [59]. This.voi file was used to measure the location (MNI coordinates) and volume (in ml) of individual lesions and to create within group overlaps of multiple lesions using the MRIcron program.

### Experimental procedures

Participants were asked to make a series of choices between two risky gambles alone (Solo trials) and after observing others’ choices (Info trials) (Fig 1A) [8]. In each pair of gambles, one gamble was always riskier than the other gamble; the two gambles had the same high- and low- payoff probabilities, but the riskier gamble had greater payoff variance. Participants were instructed that they were playing within a group of five others and that two other players’ choices would be randomly selected and shown on some trials. To ensure participants’ understanding, a brief quiz was administered following the task instruction and any erroneous answers were addressed. Unbeknownst to participants, for the purpose of the current study, Info trials were drawn without replacement from a uniform distribution that comprised equal numbers of trials where both other players chose the safe gamble (Info: ‘safe’), both others chose the risky gamble (Info: ‘risky’), or one player chose the safe and the other player chose the risky gamble (Info: ‘mix’). Gambles used in the current study were developed to optimally observe risky decision-making under social influence, as detailed in Chung *et al*. [8]. In brief, we developed eight unique lottery menus adapted from Holt and Laury [60]. For each participant, four lottery menus were randomly selected among the eight lottery menus and paired with six levels of payoff probabilities (probability of high-payoff: 40, 50, 60, 70, 80, and 90%). These 24 unique pairs of gambles (one safer and one riskier gamble) were used in each trial type (Solo; Info: ‘safe’; Info: ‘risky’; and Info: ‘mix’), so in total, each participant had 96 trials (4 lottery menus × 6 probabilities × 4 trial types). The trial order was randomized for menu × probability × trial type with a unique order per participant. Participants were informed that their final payment was not dependent on any other players’ choices and would be paid at the end of the task based on their choices. At the end of the task, one of the participants’ choices was randomly selected and carried out to determine their final payment.

We note that we have previously shown that the present task captures a social influence process (as opposed to a more general visual information effect or non-social influence). Specifically, in our previously published work [8], we introduced the task used here and also enrolled a separate cohort of N = 30 participants in a non-social condition designed to evaluate the ‘social’ nature of the social influence effect. In the non-social control task, participants were instructed that the two other ‘players’ were computers choosing between the gamble options, and the players were labeled as such on the display; all other task aspects were identical to the social version. In this non-social control, no effect of the computer chosen options on participants’ choices were observed (see S8, reprinted with permission from [8], for further information).

## Quantification and statistical analysis

### Statistical analyses

All statistical tests, except where indicated otherwise, were analyzed using a non-parametric bootstrapping method [61]. For each test, the data from all groups were drawn with replacement and assigned to groups that each had equal size as the original groups. For 10,000 iterations, the statistical test of interest was then performed on this ‘resampled’ data to obtain the null distribution. Specifically, for comparison between groups (t-tests and F-tests), a null test statistic distribution was obtained by randomly selecting data points from a set of pooled data points from all participants in each group being compared. For within-group analysis (correlation and regression tests), data were ‘unpaired’ by independently drawing the dependent and independent variables for each subject from the set of group values for that given variable. For the categorical demographics data, a chi-squared bootstrapped test was used; this test has been shown to be more accurate than the chi-square test on its own and has been suggested to be better than Fisher’s exact test which has been criticized for being overly conservative [62]. For all tests, once the null distribution of the test statistic was obtained, significance could be assessed by comparison with the actual test statistic. Specifically, test statistics whose *absolute* value was greater than 9,500 of the absolute value of the null samples were considered significant (the equivalent of a two-tailed test with alpha level of 0.05). *P* values were calculated by getting the total percentage of null samples (absolute value) whose test statistics were greater than the absolute value of the test statistic of the actual data set. All statistical analysis was done using MATLAB R2015a (Mathworks) and R 3.3.0 [63]. To test our main hypotheses, we combined the dACC and insula lesion groups. When significant differences were observed, to assess robustness of differences to individual risk preferences, we performed the same statistical test again but on the residuals of the variable of interest after regressing out individuals’ risk preference. Additionally, we proceeded with post-hoc analyses comparing dACC and insula lesion groups separately to explore the possibility of lesion-specific differences. When non-significant results were observed, we followed up with Bayesian null hypothesis testing (using the bayesFactor package in MATLAB) to quantify the extent of evidence (BF_null_) that the groups being compared were similar (for t-tests) or that the variables being compared were unrelated (for correlation tests).

### Computational modeling

The full model used in this paper is labeled as the Hybrid model. This model is a mixture of two different models: OCU-based and OCU-free models. Below, we first define these models separately, then specify the full model. Note that because the full model is a mixture model, the OCU-based and OCU-free models are both nested within this full model. We additionally use the following nested models in a formal model comparison: (i) Solo risk preference, (ii) OCU-based, and (iii) OCU-free (S4 Fig).

*1) Solo risk preference*. This model is a standard power utility model used to explain utility-based risky-decision making. We use this model to test whether insula and dACC lesion groups had disrupted utility-based risky decision-making on Solo trials. Note that this model is a nested model of the OCU model (where OCU = 0).

$$U_{safe}=P_{high}{\left(V_{high,safe}\right)}^{\alpha }+(1-P_{high}){\left(V_{low,safe}\right)}^{\alpha }$$

$$U_{risky}=P_{high}{\left(V_{high,risky}\right)}^{\alpha }+(1-P_{high}){\left(V_{low,risky}\right)}^{\alpha }$$

$$P_{safe}={\left[1+exp(-\beta \;(U_{safe}-U_{risky}))\right]}^{-1}$$

where P_high_ is the probability of high-payoff, V is the value of the high- and low- payoffs (as indicated) in the safe and risky gambles, and α is risk preference where α = 1 indicates risk neutrality, α > 1 indicates risk seeking, and 0 < α < 1 indicates risk aversion. β is the sensitivity to utility differences between the safe and risky gambles (inverse temperature) where high value indicates less noisy behavior. The model includes four free parameters (two hyperparameters [mean, variance] × two parameters): β and α.

*2) OCU-based model*. The OCU-based model adds utility (positive or negative) to the option chosen by others, and thus modifies one’s original subjective valuation of the option that was computed based on their own risk preference:

$$P_{OCU}={\left[1+exp(-\beta \;(U_{safe}-U_{risky}+\delta \;OCU))\right]}^{-1}$$

where OCU is an additional utility added to the gamble chosen by others, and δ is an indicator where δ = 1 for Info: ‘safe’ trials, δ = –1 for Info: ‘risky’ trials, and δ = 0 otherwise. The model includes six free parameters (two hyperparameters [mean, variance] × three parameters): β, α, and OCU.

*3) OCU-free model*. This model assumes that individuals have a mixed tendency to follow others’ unanimous choices, and that they use their own risk preference on Solo and Info: ‘mix’ trials (i.e., P_safe_ = [1 + exp(−β (U_safe_ − U_risky_))]^−1^).

ω_follow_: This ‘follow others’ choices’ weight captures participants’ tendency to select the same option as chosen by others (the safe or risky gamble), independent of utility computations.


$$P_{follow}=\left\{ \begin{array}{l} 1,\;on\;Info:\;‘safe’\;trials;\\ 0,\;on\;Info:\;‘risky’\;trials;\\ not\;applicable,\;on\;Solo\;and\;Info:\;‘mix’\;trials \end{array}\right.$$

ω_oppose_: This ‘oppose others’ choices’ weight captures participants’ tendency to choose the different option from the choice of others independent of utility computations.


$$P_{oppose}=\left\{ \begin{array}{l} 0,\;on\;Info:\;‘safe’\;trials;\\ 1,\;on\;Info:\;‘risky’\;trials;\\ not\;applicable,\;on\;Solo\;and\;Info:\;‘mix’\;trials \end{array}\right.$$

Note that P_follow_ and P_oppose_ are defined as probabilities of choosing the safe gamble, following and opposing others’ choices, respectively.

Two normalizing weights—ω_follow_ and ω_oppose_—determine a relative relationship. Note that ω_follow_ + ω_oppose_ = 1 in this model and that each Info: ‘safe’ and Info: ‘risky’ trial choice is assumed to arise from a combination of the mixture weights. The model includes five free parameters (two hyperparameters [mean, variance] × two parameters + two weights minus one): β, α, and one ω. Note that although there are two weights in this model, only one is considered a free parameter; the second can only take on only one value since they must add to unity.

*4) Social*: *Hybrid model*. We constructed a hybrid model of OCU-based and OCU-free models, which extends our previously suggested ‘other-conferred utility (OCU)’ model [8]. The extended mixture model uses weights, combining the three weights described above as follows:

$$P_{safe}=\left\{ \begin{array}{c} \omega_{follow}(1)+\omega_{oppose}(0)+{\;\omega }_{utility}(P_{OCU}),on\;Info:\;‘safe’\;trials;\\ \omega_{follow}(0)+{\;\omega }_{oppose}(1)+\omega_{utility}(P_{OCU}),on\;Info:\;‘risky’\;trials;\\ P_{OCU},on\;Solo\;and\;Info:\;‘mix’\;trials \end{array}\right.$$

Note that P_OCU_ on Solo and Info: ‘mix’ trials is equivalent to one’s probability of choosing the safe gamble based on utility differences between the safe and risky gambles (P(safe) = P_OCU_ = [1 + exp(−β (U_safe_− U_risky_))]^−1^). The Hybrid model includes eight free parameters (two hyperparameters [mean, variance] × three parameters + three weights minus one): inverse temperature β, risk preference α, OCU, and two ωs.

We extended this model to include the possibility that social information is used in the decision-making process independently of utility computation. This mixture model (‘Hybrid’ model) consists of three probability weights, including: (i) probability of choosing the same gamble as others independent of the gamble information, (ii) probability of choosing the different option from others independent of the option information, and (iii) probability of choosing the safe option determined based on the expected utility [24] difference between the two options after taking into account the additional utility added to the option chosen by others (OCU). Three normalizing weights—ω_follow_, ω_oppose_, and ω_utility_—determine a relative relationship. Note that ω_follow_ + ω_oppose_ + ω_utility_ = 1 and that each choice on Info: ‘safe’ and Info: ‘risky’ trials is represented as a mixture. The OCU weight (ω_utility_) specifies how much one uses social information following the OCU-based computation, while the ‘follow others’ choices’ weight (ω_follow_) specifies the extent to which one uses the OCU-free heuristic (Fig 2). Alternative models for risk and social influence are discussed in S1 Text.

### Behavioral model specifications and model comparison

For each model, unless otherwise specified, all 96 trials per participant were used for parameter estimations. Each model was constructed in a hierarchical structure where individuals were assumed to be sampled from a common group level distribution [64], aside from the weight parameters. For all hierarchical parameters, the group level distributions were defined as Gaussian with free group-level mean (μ), SD (σ), and a standard normal distribution (Normal(0, 1)) following non-centered parameterization [65]. We specified one group level distribution for each parameter across the lesion and control groups. For inverse temperature, an additional ‘group difference’ parameter was estimated separately for both the insula and dACC lesion groups, as these groups differed from NCs in their inverse temperature estimates in preliminary analyses examining Solo trials using the solo risk preference model. For inverse temperature β and risk preference α, we applied an exponential transformation. We estimated the hyperparameters (i.e., group-level priors) for β, α, and OCU using weak priors: μ ~ Normal(0, 10) and σ ~ half-Cauchy(0, 2.5). Sets of ω were defined as simplex, so that the sum of weights was always 1. We applied a group-level uniform distribution to ωs, thus the weights were only estimated at an individual-level.

For all parameter estimations, we used Markov chain Monte Carlo (MCMC) with the No-U-Turn Sampler (NUTS) [66] variant of Hamiltonian Monte Carlo implemented in Stan [67] in its R interface [65]. A total of four chains were run where each drew 5000 samples, discarding the first 2000 samples for burn-in (giving a total of 12,000 post-burn-in draws). All values of the potential scale reduction factor ($\hat{R}$) were below 1.1 and we visually inspected the chains for parameter convergence and good mixing [68].

As specified above, the Hybrid model is the most general model. That is, other models can be represented as a special case of the general model (nested models). When comparing model fits between participant groups, we calculated the median negative log likelihood (–LL). To formally compare fits between models including penalties for model complexity, we computed an integrated Bayesian Information Criteria (iBIC) [69] per model. Note that iBIC evaluates model fit not just at point estimates, but across the whole posterior distribution [69].

### Parameter comparison

Because individuals’ estimated parameters using hierarchical Bayesian estimation are dependent on the group posterior, to allow for group differences to be identified (if they are present), we estimated two additional group-level mean difference parameters for both the inverse temperature and risk preference parameters; statistical tests were conducted by examining whether or not the 95% credible interval (CI) for each group-level parameter crosses zero. Because we found inverse temperature, but not risk preference, differences between groups in the Solo trials, all models presented here (unless otherwise specified) were estimated using an inverse temperature mean difference parameter for each lesion group.

Bootstrapped t-tests were used to compare weights among groups. Note that the weights are not dependent on the group distribution (because they were not part of a hierarchical structure), and thus, classical t-tests can be used to examine individual differences (Fig 3A–3C). To examine the correlation between model fit (−LL) and ω_follow_, −LL was estimated from the Solo risk preference model using only Solo trials and ω_follow_ was estimated from the Hybrid model (Fig 3D).

### Parameter recovery

To confirm that the task design is sensitive enough to capture individual differences within the model structure, we sampled 44 sets (the same as our sample size) of parameters (α and OCU for 44 simulated subjects; ‘true parameters’ hereafter) using the mean and standard deviation for the estimated group-level distributions (estimated from individuals’ raw choice data). Actual β parameters were used from each subject. For the three weights (ω_follow_, ω_oppose_, and ω_utility_), we sampled the weights uniformly from a unit simplex, satisfying ω_follow_ + ω_oppose_ + ω_utility_ = 1 and restricting values between minimum and maximum values of subject ωs. Using the same gamble sequences that were presented to participants, we simulated individual choice data, and examined whether the true parameters were recovered or not. Because simulated choices are subject to some randomness, some simulated choice sets generated sequences that did not meet inclusion criteria for the study. As specified earlier (in **Participants**), this can lead to estimation issues. Thus, simulated behaviors went through the same exclusionary criteria as actual participants’ behavior (see **Participants** for exclusion criteria). If a simulated participant’s behavior was excluded, a new set of parameters was generated and used to re-simulate behavioral choices. The same parameter estimation procedure was used as described above. All estimated parameters were significantly correlated with the true parameters used to generate simulated data (inverse temperature (log transform): Pearson’s r = 0.81, *P* = 1.86e−11; risk preference: Pearson’s r = 0.86, *P* = 7.32e−14; OCU_normalized_: Pearson’s r = 0.76, *P* = 2.75e−09; ω_follow_: Pearson’s r = 0.88, *P* = 4.38e−15; ω_oppose_: Pearson’s r = 0.90, *P* = 1.46e−16; ω_utility_: Pearson’s r = 0.73, *P* = 2.44e−08). Note that the OCU parameter was normalized to show the actual contribution of OCU to the utility function, as follows:

$$OCU_{normalized}={\left[1+exp(-\beta \times OCU)\right]}^{-1}.$$

Stan makes it possible to efficiently estimate models with multiple hierarchical levels and many parameters. It does so by using a Hamiltonian Monte Carlo algorithm to move quickly through the posterior distribution with minimal correlation between parameters. To efficiently sample from the posterior distribution, it simulates a Hamiltonian ‘trajectory.’ Comparing the initial and final Hamiltonian values in a given iteration provides a straightforward way to detect potential sampling errors. Specifically, these are detected when Stan’s approximation of a Hamiltonian trajectory deviates significantly from the true Hamiltonian trajectory, called a ‘divergent transition.’ In practice, when these ‘divergent transitions’ occur, it can bias the posterior distribution. To be clear, none of the models estimated on participant data had any divergent transitions. However, in model recovery for the Hybrid model, approximately 1.05% of iterations had divergent transitions. Because we know the actual estimates that the simulated data was generated from, we can directly test if the divergent transitions biased the recovery results. To determine this, we compare the estimates to the true parameters. By definition, if the divergent transitions bias the posterior, it would push the estimates away from the true parameters. As we see strong correlations between the true parameters and parameter estimates from simulated data, we conclude that the divergent transitions did not bias the posterior estimates *and* that the model is sensitive enough to capture individual differences within the model structure. Similar model estimation issues and logic apply to the model recovery (S4) and exclusion criteria analyses (S7).

### Model recovery

In addition to parameter recovery, we performed model recovery to assess whether the four models used and tested in this paper are dissociable [70]. To do so, we simulated participant choices using each of the four models: Hybrid, Solo risk preference, OCU-based, and OCU-free. We simulated choice data according to each model for 44 subjects (referred to as ‘Simulated model’ hereafter). These data were created based on parameters drawn from the actual distribution of estimated parameters for each model, restricted to be within the range of parameter estimates. Actual β parameters were used for each model (in the same way as **Parameter recovery**). The data generated from each Simulated model was then used to fit all four models. We then calculated how well each of these models (Fit model) fit the data using iBIC. The fits are calculated at the group level as total iBIC (S4a) and at an individual level using participant counts of best fit for each model, calculated as a proportion summing to 100% for each Simulated model to calculate a confusion matrix (S4b) or calculated as a proportion summing to 100% for each Fit model to calculate the inversion matrix (S4c). We note that these analyses indicate that these models are not well-dissociable. Thus, while our main results are based on robust parameter estimates of the Hybrid model, it is important to keep in mind that the Hybrid model alone is unlikely to fully and uniquely capture the range of social influences on risky decision-making. See S4 for further discussion of this point.

## Supporting information

S1 Fig Individuals with insula and dACC lesions show impairments in risky decision-making. **(a)** We used multiple linear regression to examine whether the lesion group was indeed impaired in risky decision-making. Specifically, we set group identity (NC vs lesion), probability of winning the high payoff, and their interaction as predictors of individuals’ gamble choices (proportion of safe choices, P(safe)) on Solo trials. As expected, on average, across all groups, individuals chose the safe option significantly less as the probability of winning the high payoff increased (*P* = 9.2e−4). In addition, the interaction effect was significant (group (NC vs lesion) × probability interaction: *P* = 0.022). Specifically, the extent to which individuals chose the safe option less as a function of the gamble’ probability was attenuated in the lesion group compared to NCs. In line with previous reports [20,21], these results suggest that individuals with insula and dACC lesions are impaired in risky decision-making. **(b)** Lesion participants and NC response times in Solo trials were compared. Individuals with insula or dACC lesions took significantly longer to make a choice compared to NCs (*P* = 0.026; NC vs insula: *P* = 0.045; NC vs dACC: *P* = 0.066), which provides an additional measure indicating disrupted decision-making about risky options. Each point represents an individual participant; Error bars represent s.e.m.
(PDF)

S2 Fig Visualization, parameter recovery, and behavioral validation of the Hybrid model. **(a)** To confirm that we can identify each parameter independently from other parameters within the Hybrid model, we conducted a parameter recovery analysis. To do so, we simulated artificial data and estimated the model on that data to see if the ‘true parameters’ used to simulate data could be identified. Correlations between true parameters and estimates indicate parameter recovery for that specific parameter (see Materials and Methods for parameter recovery procedure). All parameters included in the model showed positive correlation between the true and estimated parameters, indicating that the Hybrid model could be recovered: inverse temperature (log transform; Pearson’s r = 0.81, P = 1.86e–11), risk preference (Pearson’s r = 0.86, *P* = 7.32e–14), OCU_normalized_ (Pearson’s r = 0.76, *P* = 2.75e–09), ω_follow_ (Pearson’s r = 0.88, *P* = 4.38e–15), ω_oppose_ (Pearson’s r = 0.90, *P* = 1.46e–16), and ω_utility_ (Pearson’s r = 0.73, *P* = 2.44e–08). **(b-d)** To confirm that the ω_follow_ and ω_oppose_ parameters are capturing an individual’s behavior, we compared these parameters to a model-agnostic measure of an individual’s tendency to conform to or oppose the choices of others. In healthy adults, we previously showed individuals who were more risk averse (or seeking) were more likely to conform when others chose the safe (or risky) choices but less likely to conform when others chose the risky (safe) choices. Moreover, in these individuals utilizing the OCU-based model, the tendency to conform to others’ safe choices was negatively correlated with the tendency to conform to others’ risky choices. Individuals following this pattern will fall close to the y = 1 − x line in **b**. Conversely, individuals who tend to use the OCU-free weights to a greater extent in the decision-making process will fall further away from this line, since they are more likely to follow or oppose others regardless of the type of information. Thus, to validate our model, we test if the orthogonal distance from the y = 1 − x line (the solid lines in **b** are shown as examples) correlates with the extent to which individuals use the OCU-free weights. As expected, there is a significant correlation between this model-agnostic measure of conformity tendency and both ω_follow_ (**c**; r = 0.72, *P* = 4.07e−08, robust correlation) and ω_oppose_ (**d**, r = −0.77, *P* = 1.33e−09, robust correlation) across all individuals, which shows that the model parameters accurately capture individuals’ behavioral choices. Each point represents an individual participant, and lines are the regressions between the indicated parameters.
(PDF)

S3 Fig Individual model fits and probability normalizing weights. **(a-c)** Each individual’s behavioral choices on Info: ‘safe’ (blue) and Info: ‘risky’ (red) trials were overlaid with their predicted choices (solid lines) based on the Hybrid model. For each participant, P(safe) is binned and averaged based on the expected value difference between the safe and risky gambles. **(d-f)** Estimates of normalizing weights between decision computations are shown for each group. While there existed individual variation across groups, a majority of those with dACC or insula lesions showed greater ω_follow_ weights than OCU weights (ω_utility_). Estimates shown are from the Hybrid model (see Materials and Methods for parameter estimation details).
(PDF)

S4 Fig Model recovery analysis. To evaluate whether our models are dissociable from one another [70], we performed model recovery analyses. Specifically, we simulated choice data from each model (Simulated model; Hybrid, Solo risk preference, OCU-based, OCU-free) and tested which model best fit the simulated data (Fit model). **(a)** At the group level, the best fit for each simulated model was the true model used to generate the data. **(b)** At the individual level, we examined the best fitting model for each simulated participant. As expected, the model used to generate the data had the highest proportion of simulated participants as its best fit. **(c)** We also calculated the inversion matrix (i.e., the proportion of the time that the best fitting model was generated by the simulated model). For all four models, the best fitting model matches the simulated data the largest proportion of the time. However, note that these models are not well-dissociable (i.e., the confusion and inversion matrices differ from the identity matrix). This could be due to several reasons, including the nested nature of the models, a relatively small contribution of the OCU parameter to behavior (as compared to the heuristic weights or risk preference), and/or the presence of multiple overlapping processes during socially-situated decision making about risky options, among other factors. While our main results are based on robust parameter estimates of the Hybrid model (and not model fit), it is important to keep in mind that the Hybrid model alone is unlikely to fully and uniquely capture the range of social influences on risky decision-making.
(PDF)

S5 Fig Formal model comparison and result consistency across models and replicability across subject groups. **(a)** The model fit of the Hybrid model was compared with other nested models. The Hybrid model (see Materials and Methods for model structure) explained participants’ behavioral choices the best (smaller integrated Bayesian information criteria (iBIC) indicates better fit). Note that we performed a parameter recovery analysis (S2) and showed that we could separately identify each parameter. **(b)** In an independent sample of healthy controls (N = 57, [8]), we show the same relationship (as in Fig 2d) between non-social model fit (–LL) and individuals’ social conformity heuristic (Pearson’s r = 0.51, *P* = 5.78e–05, robust correlation) as was found in the subjects in the current study. Weight parameters for social conformity heuristic were estimated using the Hybrid model, while model fit was calculated from the Solo risk preference model only using Solo trials. Models for healthy controls were estimated in a similar fashion as the original study participants, with the exception that inverse temperature was constrained between 0 and 50, and risk preference was constrained between 0 and 2. Each point represents an individual participant, and lines are the regressions between the indicated parameters; Error bars represent s.e.m.
(PDF)

S6 Fig Others’ choices from previous trials do not influence subsequent Solo trial choices. To examine whether the effects of social influence from the past trials persist, we compared Solo trial choices with and without Info trials either on one or two trials back. Specifically, we estimated two mixed-effects logistic regression models (using subject as the random effect) separately for NCs and the lesion group to predict safe choices on Solo trials with a regressor corresponding to previous social influence (Info: ‘safe’ trials were coded as 1, Info: ‘risky’ trials were coded as −1). These analyses showed that there was no effect of previous social influence (neither one- nor two- back) on the choices made on subsequent Solo trials in either NCs or the lesion group (all *P*s > 0.05). The fixed effect beta coefficients and their standard errors are depicted for the one- and two- back conditions, and for each group separately.
(PDF)

S7 Fig Main results remain consistent across different exclusion criteria. In the main text, we use two main exclusion criteria: 1) participants who chose the option with the greater high payoff value less frequently as the probability of winning increased, suggesting a misunderstanding or lack of attention to the task, and 2) participants who always chose either the safe or the risky option in the Solo trials, and thus for whom bi-directional influence is not possible. To check the robustness of our main findings with respect to exclusions, we conducted the main analyses without exclusions; these analyses indicate that our main results remain largely consistent even when individuals excluded from our analyses in the main text are reinserted. **(a-c)** When all participants were included (control N = 46, insula N = 11, and dACC N = 11), we observed a trending result of the worse model fit (*P* = 0.12) and the increased follow weight (*P* = 0.11) for lesion participants. In addition, the correlation between worse model fit and the follow weight was significant (r = 0.45, *P* = 1.2e−04). **(d-f)** When participants who did not show reasonable choices in trivial cases remained excluded, but the ones who always chose either the safe or risky option were reincluded (control N = 25, insula N = 10, and dACC N = 8), we observed a trending result of the worse model fit (*P* = 0.092) and the increased follow weight (*P* = 0.027) for lesion participants. Furthermore, the correlation between worse model fit and the follow weight was significant (r = 0.46, *P* = 4.9e−04). Overall, although some of the results are statistically marginal, these data show that the main results remain consistent even when we are more lenient with our exclusion criteria.
(PDF)

S8 Fig (Reproduced from Chung et al., [8] with permission) Computer-generated other options have no influence on participants’ choices. As a control analysis for the 2015 study introducing this task [8], we performed a ‘computer control’ on 30 healthy participants. Specifically, to assess whether the observed influence of others was a social or more general information effect (e.g., priming with visual information), we implemented a separate behavioral experiment instructing participants that ‘Info’ trials were computer-generated choices. The visual aspects and trial structure of the original game were maintained, and as in the original task, participants chose between two gambles. Participants were instructed that on some trials (previously the ‘Info’ trials), prior to the participant’s decision, two computers would randomly pick among the options, and these two options would be presented (‘Computer Info’ trials). As in the original experiment, ‘Solo’ trials were interspersed with the Computer Info trials. No influence of computer-selected options on participants’ choices was observed (repeated measures ANOVA, F(3, 87) = 0.71, P = 0.55; paired t-tests: Safe vs Solo, t(29) = 0.61, P = 0.55; Mix vs Solo, t(29) = 0.90, P = 0.37; Risky vs Solo, t(29) = -0.52, P = 0.37). Error bars show s.e.m.
(PDF)

S1 Text Informal comparison with alternate risky decision-making and social influence models. (DOCX)
