# Supplementary figures and images for: Social conformity is a heuristic when individual risky decision-making is disrupted
Source: PLoS Comput Biol. 2024 Dec 2;20(12):e1012602. doi: 10.1371/journal.pcbi.1012602 (PMC11651703; doi:10.1371/journal.pcbi.1012602)

**a**

P(safe) on Solo trials

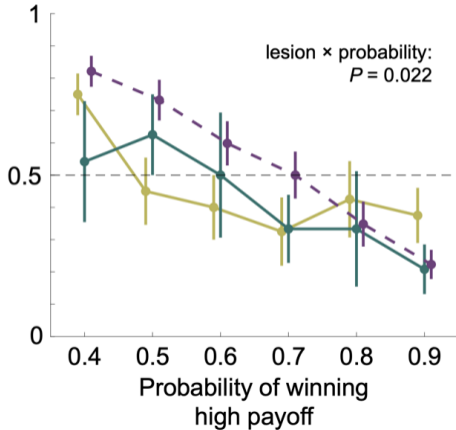

**b**

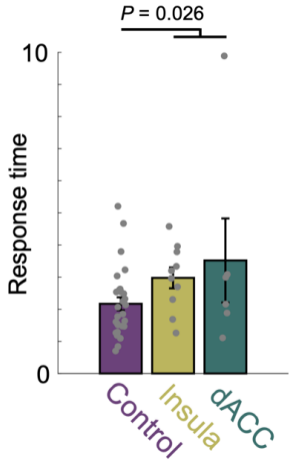

Supplement: S1 Fig — (a) We used multiple linear regression to examine whether the lesion group was indeed impaired in risky decision-making. Specifically, we set group identity (NC vs lesion), probability of winning the high payoff, and their interaction as predictors of individuals’ gamble choices (proportion of safe choices, P(safe)) on Solo trials. As expected, on average, across all groups, individuals chose the safe option significantly less as the probability of winning the high payoff increased (P = 9.2e−4). In addition, the interaction effect was significant (group (NC vs lesion) × probability interaction: P = 0.022). Specifically, the extent to which individuals chose the safe option less as a function of the gamble’ probability was attenuated in the lesion group compared to NCs. In line with previous reports [20,21], these results suggest that individuals with insula and dACC lesions are impaired in risky decision-making. (b) Lesion participants and NC response times in Solo trials were compared. Individuals with insula or dACC lesions took significantly longer to make a choice compared to NCs (P = 0.026; NC vs insula: P = 0.045; NC vs dACC: P = 0.066), which provides an additional measure indicating disrupted decision-making about risky options. Each point represents an individual participant; Error bars represent s.e.m. (PDF) [file pcbi.1012602.s001.pdf]

**a**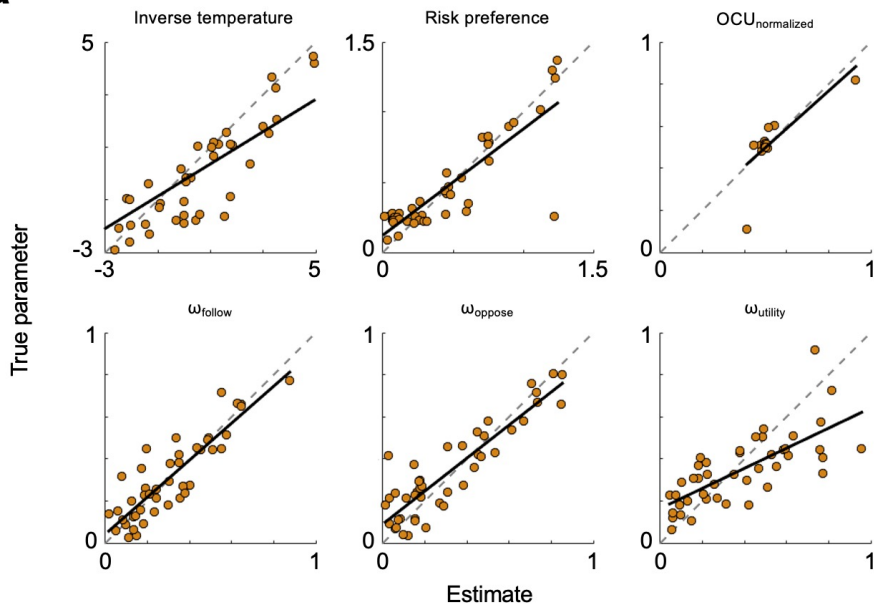**b**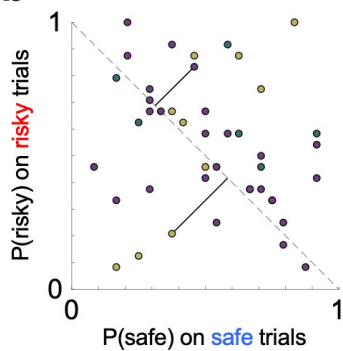**c**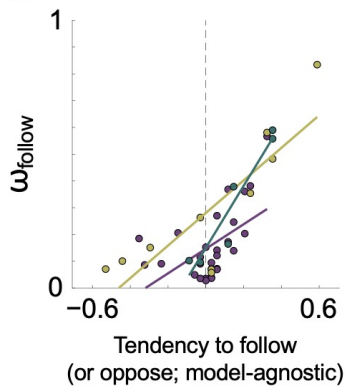**d**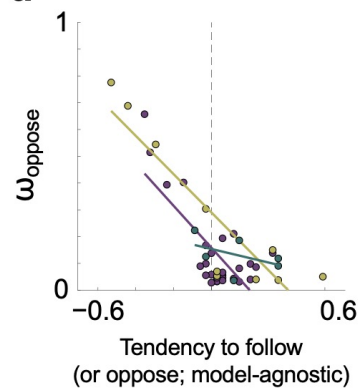

Supplement: S2 Fig — (a) To confirm that we can identify each parameter independently from other parameters within the Hybrid model, we conducted a parameter recovery analysis. To do so, we simulated artificial data and estimated the model on that data to see if the ‘true parameters’ used to simulate data could be identified. Correlations between true parameters and estimates indicate parameter recovery for that specific parameter (see Materials and Methods for parameter recovery procedure). All parameters included in the model showed positive correlation between the true and estimated parameters, indicating that the Hybrid model could be recovered: inverse temperature (log transform; Pearson’s r = 0.81, P = 1.86e–11), risk preference (Pearson’s r = 0.86, P = 7.32e–14), OCUnormalized (Pearson’s r = 0.76, P = 2.75e–09), ωfollow (Pearson’s r = 0.88, P = 4.38e–15), ωoppose (Pearson’s r = 0.90, P = 1.46e–16), and ωutility (Pearson’s r = 0.73, P = 2.44e–08). (b-d) To confirm that the ωfollow and ωoppose parameters are capturing an individual’s behavior, we compared these parameters to a model-agnostic measure of an individual’s tendency to conform to or oppose the choices of others. In healthy adults, we previously showed individuals who were more risk averse (or seeking) were more likely to conform when others chose the safe (or risky) choices but less likely to conform when others chose the risky (safe) choices. Moreover, in these individuals utilizing the OCU-based model, the tendency to conform to others’ safe choices was negatively correlated with the tendency to conform to others’ risky choices. Individuals following this pattern will fall close to the y = 1 − x line in b. Conversely, individuals who tend to use the OCU-free weights to a greater extent in the decision-making process will fall further away from this line, since they are more likely to follow or oppose others regardless of the type of information. Thus, to validate our model, we test if the orthogonal distance from the y [file pcbi.1012602.s002.pdf]

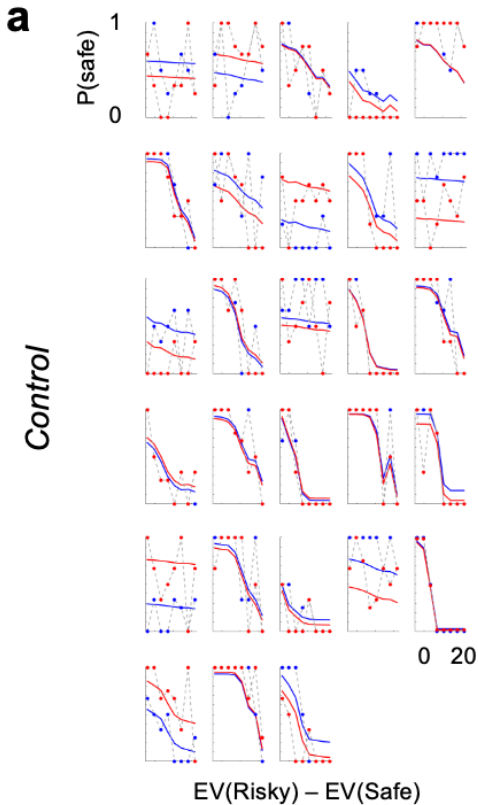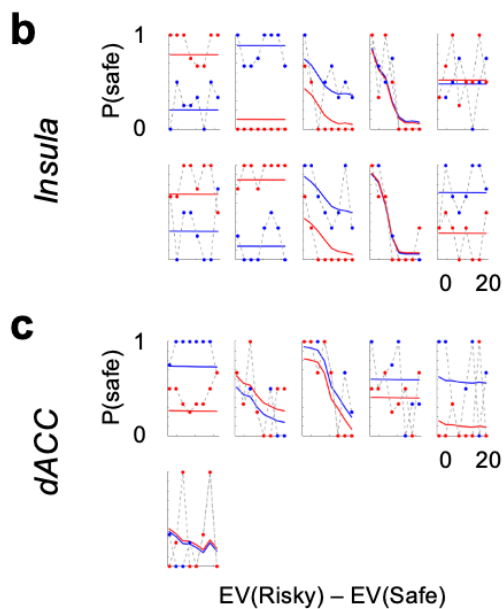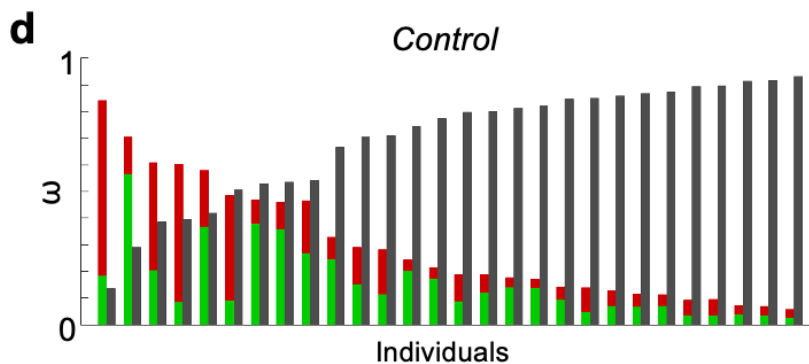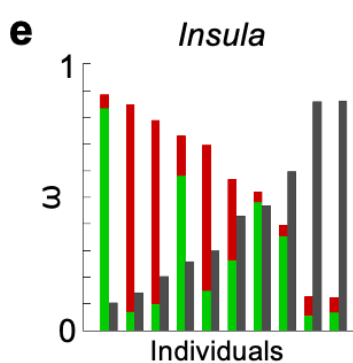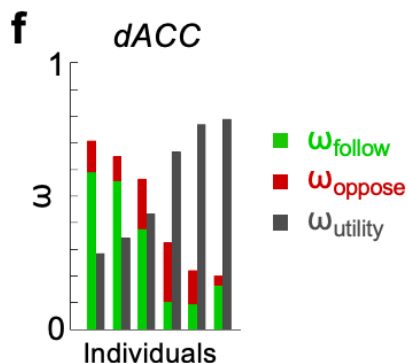

Supplement: S3 Fig — (a-c) Each individual’s behavioral choices on Info: ‘safe’ (blue) and Info: ‘risky’ (red) trials were overlaid with their predicted choices (solid lines) based on the Hybrid model. For each participant, P(safe) is binned and averaged based on the expected value difference between the safe and risky gambles. (d-f) Estimates of normalizing weights between decision computations are shown for each group. While there existed individual variation across groups, a majority of those with dACC or insula lesions showed greater ωfollow weights than OCU weights (ωutility). Estimates shown are from the Hybrid model (see Materials and Methods for parameter estimation details). (PDF) [file pcbi.1012602.s003.pdf]

**a**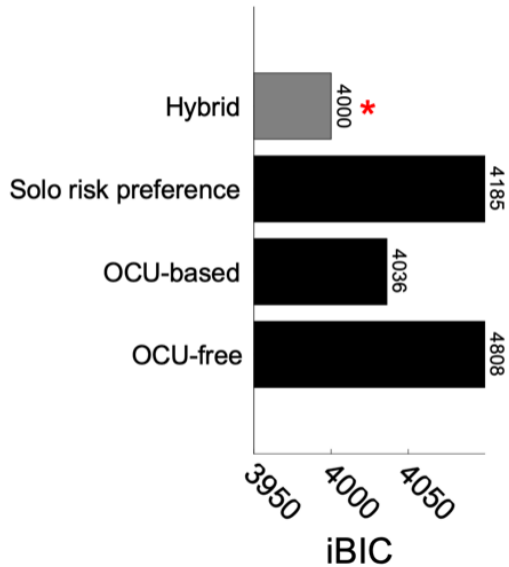**b**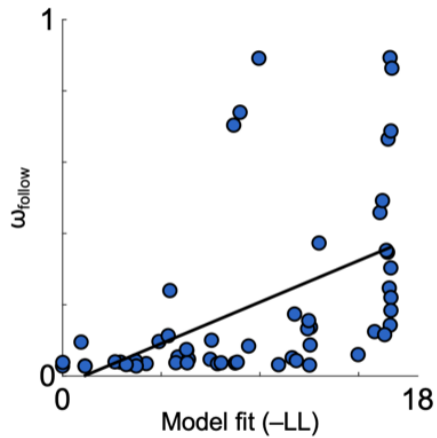

Supplement: S5 Fig — (a) The model fit of the Hybrid model was compared with other nested models. The Hybrid model (see Materials and Methods for model structure) explained participants’ behavioral choices the best (smaller integrated Bayesian information criteria (iBIC) indicates better fit). Note that we performed a parameter recovery analysis (S2) and showed that we could separately identify each parameter. (b) In an independent sample of healthy controls (N = 57, [8]), we show the same relationship (as in Fig 2d) between non-social model fit (–LL) and individuals’ social conformity heuristic (Pearson’s r = 0.51, P = 5.78e–05, robust correlation) as was found in the subjects in the current study. Weight parameters for social conformity heuristic were estimated using the Hybrid model, while model fit was calculated from the Solo risk preference model only using Solo trials. Models for healthy controls were estimated in a similar fashion as the original study participants, with the exception that inverse temperature was constrained between 0 and 50, and risk preference was constrained between 0 and 2. Each point represents an individual participant, and lines are the regressions between the indicated parameters; Error bars represent s.e.m. (PDF) [file pcbi.1012602.s005.pdf]

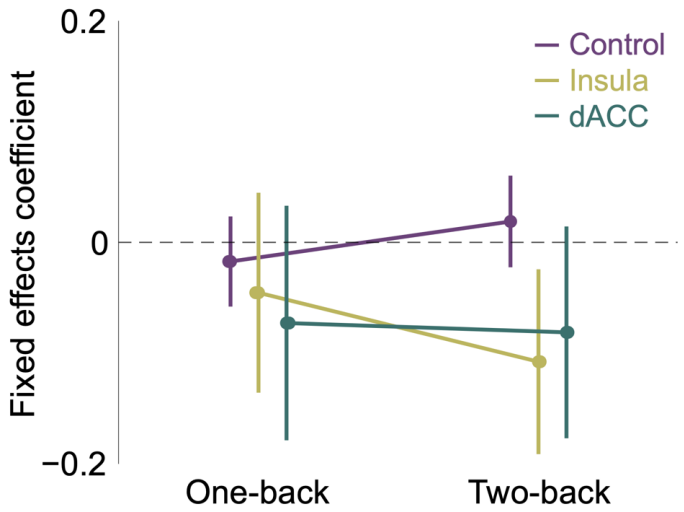

Supplement: S6 Fig — To examine whether the effects of social influence from the past trials persist, we compared Solo trial choices with and without Info trials either on one or two trials back. Specifically, we estimated two mixed-effects logistic regression models (using subject as the random effect) separately for NCs and the lesion group to predict safe choices on Solo trials with a regressor corresponding to previous social influence (Info: ‘safe’ trials were coded as 1, Info: ‘risky’ trials were coded as −1). These analyses showed that there was no effect of previous social influence (neither one- nor two- back) on the choices made on subsequent Solo trials in either NCs or the lesion group (all Ps > 0.05). The fixed effect beta coefficients and their standard errors are depicted for the one- and two- back conditions, and for each group separately. (PDF) [file pcbi.1012602.s006.pdf]

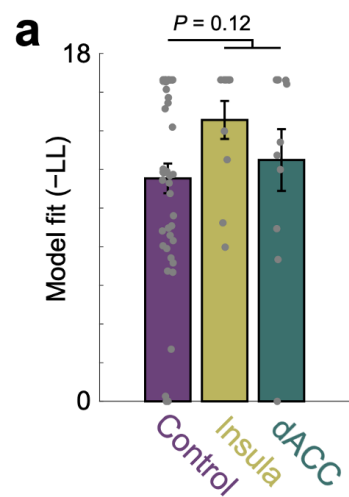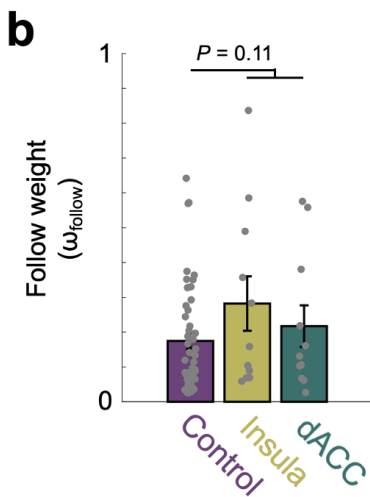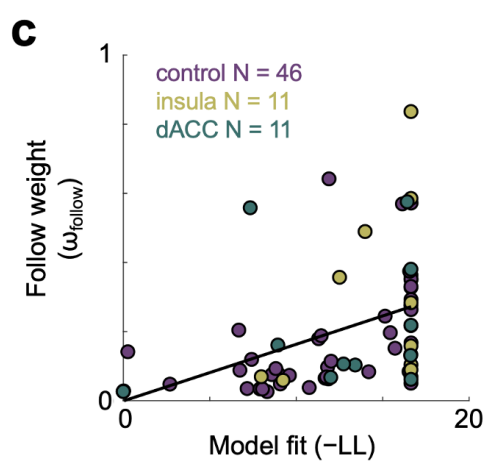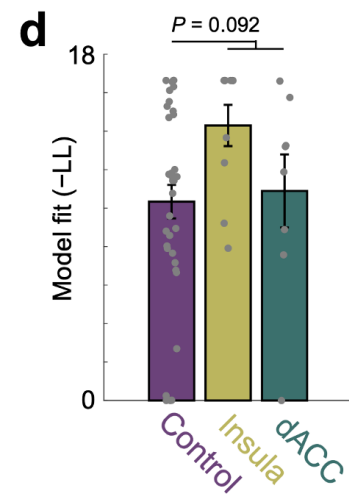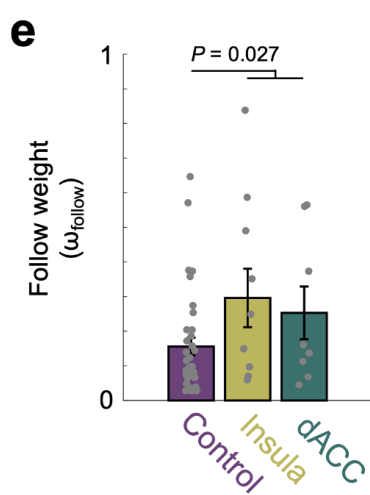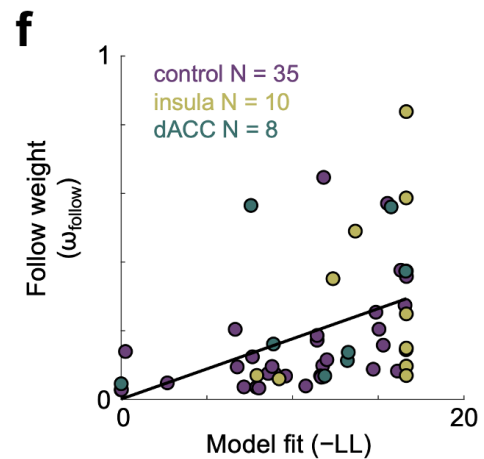

Supplement: S7 Fig — In the main text, we use two main exclusion criteria: 1) participants who chose the option with the greater high payoff value less frequently as the probability of winning increased, suggesting a misunderstanding or lack of attention to the task, and 2) participants who always chose either the safe or the risky option in the Solo trials, and thus for whom bi-directional influence is not possible. To check the robustness of our main findings with respect to exclusions, we conducted the main analyses without exclusions; these analyses indicate that our main results remain largely consistent even when individuals excluded from our analyses in the main text are reinserted. (a-c) When all participants were included (control N = 46, insula N = 11, and dACC N = 11), we observed a trending result of the worse model fit (P = 0.12) and the increased follow weight (P = 0.11) for lesion participants. In addition, the correlation between worse model fit and the follow weight was significant (r = 0.45, P = 1.2e−04). (d-f) When participants who did not show reasonable choices in trivial cases remained excluded, but the ones who always chose either the safe or risky option were reincluded (control N = 25, insula N = 10, and dACC N = 8), we observed a trending result of the worse model fit (P = 0.092) and the increased follow weight (P = 0.027) for lesion participants. Furthermore, the correlation between worse model fit and the follow weight was significant (r = 0.46, P = 4.9e−04). Overall, although some of the results are statistically marginal, these data show that the main results remain consistent even when we are more lenient with our exclusion criteria. (PDF) [file pcbi.1012602.s007.pdf]

Percent safe choices  
in COM INFO – SOLO trials

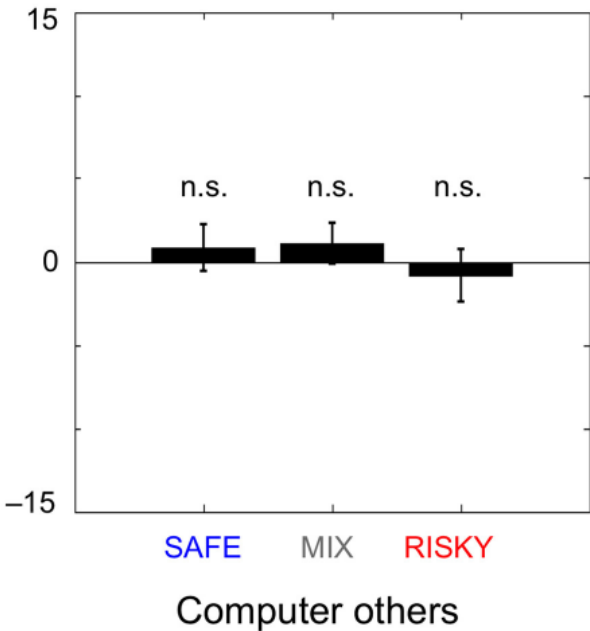

Supplement: S8 Fig — As a control analysis for the 2015 study introducing this task [8], we performed a ‘computer control’ on 30 healthy participants. Specifically, to assess whether the observed influence of others was a social or more general information effect (e.g., priming with visual information), we implemented a separate behavioral experiment instructing participants that ‘Info’ trials were computer-generated choices. The visual aspects and trial structure of the original game were maintained, and as in the original task, participants chose between two gambles. Participants were instructed that on some trials (previously the ‘Info’ trials), prior to the participant’s decision, two computers would randomly pick among the options, and these two options would be presented (‘Computer Info’ trials). As in the original experiment, ‘Solo’ trials were interspersed with the Computer Info trials. No influence of computer-selected options on participants’ choices was observed (repeated measures ANOVA, F(3, 87) = 0.71, P = 0.55; paired t-tests: Safe vs Solo, t(29) = 0.61, P = 0.55; Mix vs Solo, t(29) = 0.90, P = 0.37; Risky vs Solo, t(29) = -0.52, P = 0.37). Error bars show s.e.m. (PDF) [file pcbi.1012602.s008.pdf]
