# Supplementary material for: Social conformity is a heuristic when individual risky decision-making is disrupted
Source: PLoS Comput Biol. 2024 Dec 2;20(12):e1012602. doi: 10.1371/journal.pcbi.1012602 (PMC11651703; doi:10.1371/journal.pcbi.1012602)
Supplement: S4 Fig — To evaluate whether our models are dissociable from one another [70], we performed model recovery analyses. Specifically, we simulated choice data from each model (Simulated model; Hybrid, Solo risk preference, OCU-based, OCU-free) and tested which model best fit the simulated data (Fit model). (a) At the group level, the best fit for each simulated model was the true model used to generate the data. (b) At the individual level, we examined the best fitting model for each simulated participant. As expected, the model used to generate the data had the highest proportion of simulated participants as its best fit. (c) We also calculated the inversion matrix (i.e., the proportion of the time that the best fitting model was generated by the simulated model). For all four models, the best fitting model matches the simulated data the largest proportion of the time. However, note that these models are not well-dissociable (i.e., the confusion and inversion matrices differ from the identity matrix). This could be due to several reasons, including the nested nature of the models, a relatively small contribution of the OCU parameter to behavior (as compared to the heuristic weights or risk preference), and/or the presence of multiple overlapping processes during socially-situated decision making about risky options, among other factors. While our main results are based on robust parameter estimates of the Hybrid model (and not model fit), it is important to keep in mind that the Hybrid model alone is unlikely to fully and uniquely capture the range of social influences on risky decision-making. (PDF) [file pcbi.1012602.s004.pdf]

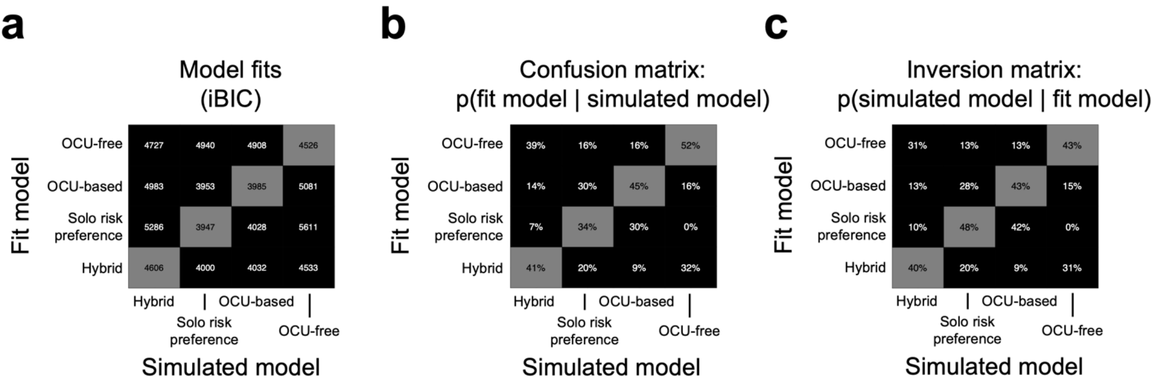
a
Model fits
(iBIC)
Fit model
OCU-free
OCU-based
Solo risk preference
Hybrid
4727 4940 4908 4526
4983 3953 3985 5081
5286 3947 4028 5611
4606 4000 4032 4533
Hybrid
Solo risk preference
OCU-based
OCU-free
Simulated model
b
Confusion matrix:
p(fit model | simulated model)
Fit model
OCU-free
OCU-based
Solo risk preference
Hybrid
39% 16% 16% 52%
14% 30% 45% 16%
7% 34% 30% 0%
41% 20% 9% 32%
Hybrid
Solo risk preference
OCU-based
OCU-free
Simulated model
c
Inversion matrix:
p(simulated model | fit model)
Fit model
OCU-free
OCU-based
Solo risk preference
Hybrid
31% 13% 13% 43%
13% 28% 43% 15%
10% 48% 42% 0%
40% 20% 9% 31%
Hybrid
Solo risk preference
OCU-based
OCU-free
Simulated model
