# Supplementary material for: Social conformity is a heuristic when individual risky decision-making is disrupted
Source: PLoS Comput Biol. 2024 Dec 2;20(12):e1012602. doi: 10.1371/journal.pcbi.1012602 (PMC11651703; doi:10.1371/journal.pcbi.1012602)
Supplement: S1 Text — (DOCX) [file pcbi.1012602.s009.docx]

**S1 Text. Informal comparison with alternate risky decision-making and social influence models.** We performed two additional exploratory analyses testing alternative models of risky decision-making and social influence. Specifically, we tested whether the mean-variance model [s1] provides a better fit to individuals’ risky choices in Solo trials and whether the preference shift model [11] better explains participants choices in Info: ‘safe’ and Info: ‘risky’ trials. As we have previously observed in other types of risky decision-making tasks in the lab, the exponential risk preference model used here provides a better fit to the data than the mean-variance model, although note that it still remains an open question which model best represents computations in the brain [54]. We also show that the OCU model used in this paper for social influence in risky decision-making better represents the present data. This is in contrast with what was found in Suzuki et al. [11]. We (and Suzuki et al. [11]) think this is likely due to a difference in task design structures, whereby the task presented in this paper uses a trial-by-trial design while Suzuki et al. [11] use a block design to induce a more long-lasting rather than transient social influence effect. Note that the mean-variance and preference shift models had some divergent transitions, and the preference shift model had some R̂ values > 1.1, thus these results should only be interpreted as exploratory. Further experiments designed to test these comparisons are necessary to be confident about these conclusions.

s1. Markowitz H. Portfolio Selection. J Finance. 1952;7: 77. doi:10.2307/2975974
